# Supplementary material for: Network Biology Analyses and Dynamic Modeling of Gene Regulatory Networks under Drought Stress Reveal Major Transcriptional Regulators in Arabidopsis
Source: Int J Mol Sci. 2023 Apr 16;24(8):7349. doi: 10.3390/ijms24087349 (PMC10139068; doi:10.3390/ijms24087349)
Supplement: Supplementary file 1 [file ijms-24-07349-s001.zip › Supplementary_Material_Kumar et al.pdf]

Supplementary Material

Supplementary Figures

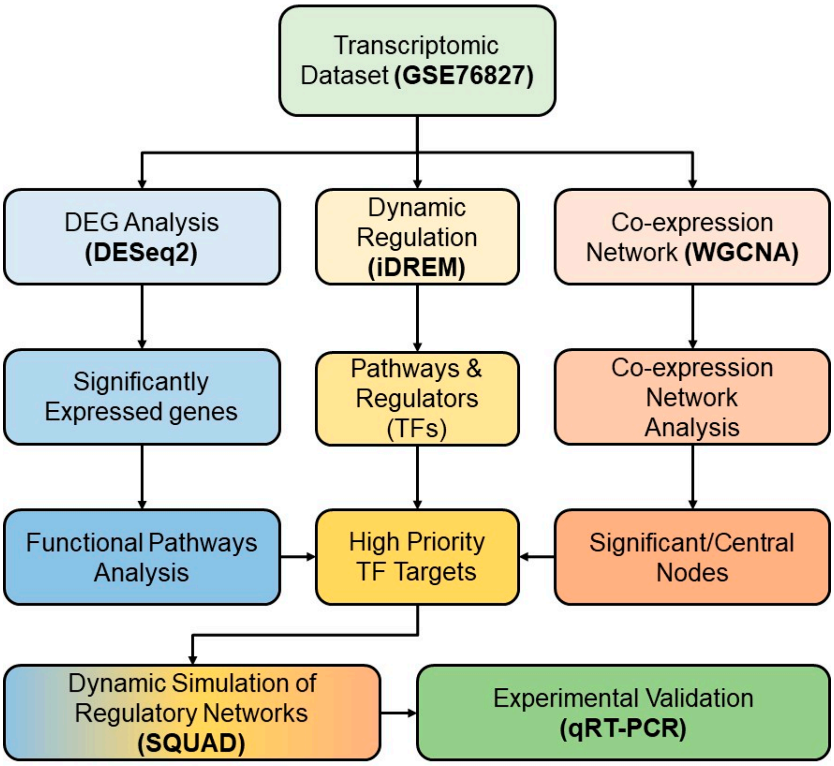

Figure S1. The pipeline describing the discovery of novel master regulators in drought.

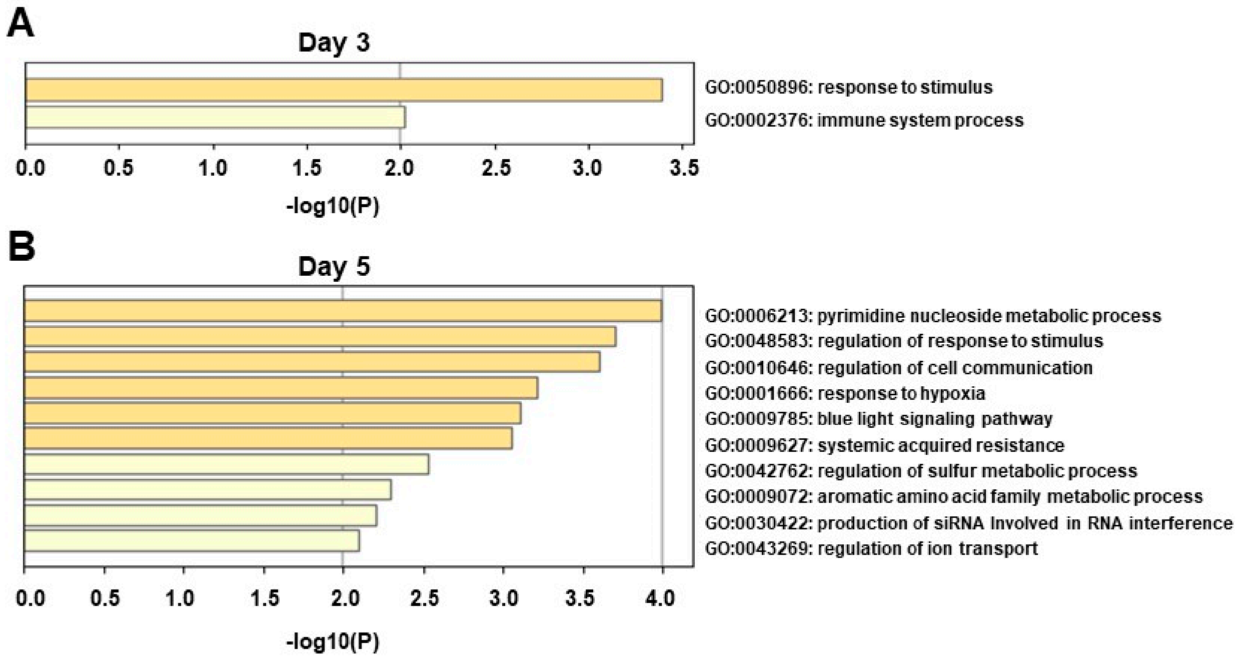

Figure S2. The gene ontology enrichment analysis of Arabidopsis unique DEGs on each (Day 3 and Day 5) after drought stress (p-value ≤0.05).

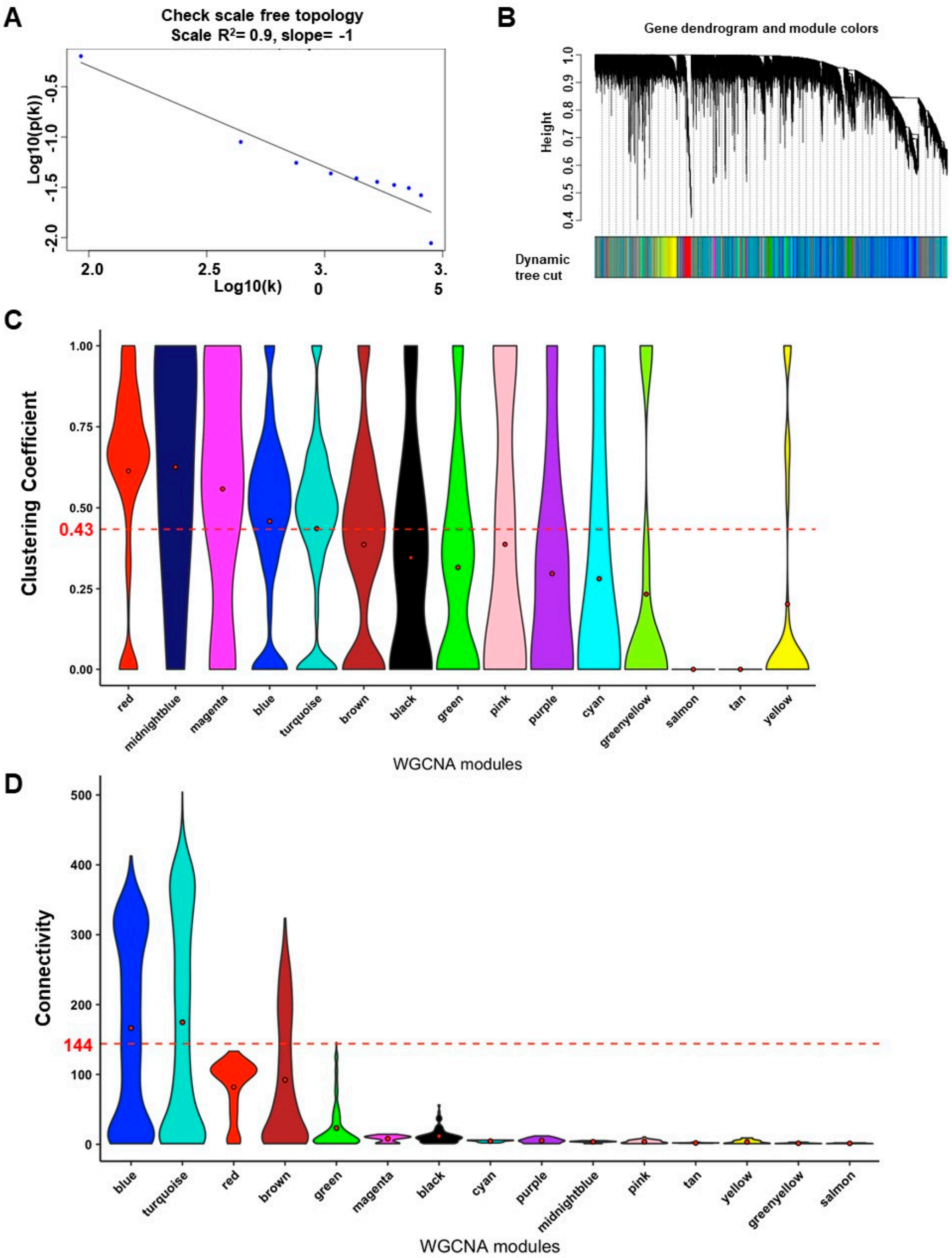

Figure S3. (A, B) The weighted gene co-expression network analysis (WGCNA) parameters for Arabidopsis drought transcriptome analysis and module identification. **(C, D)** The violin plot of clustering coefficient and connectivity to identify the most significant modules of ADGCN based on the cutoff of an average value of 0.43 and 144 for clustering coefficient and connectivity, respectively.

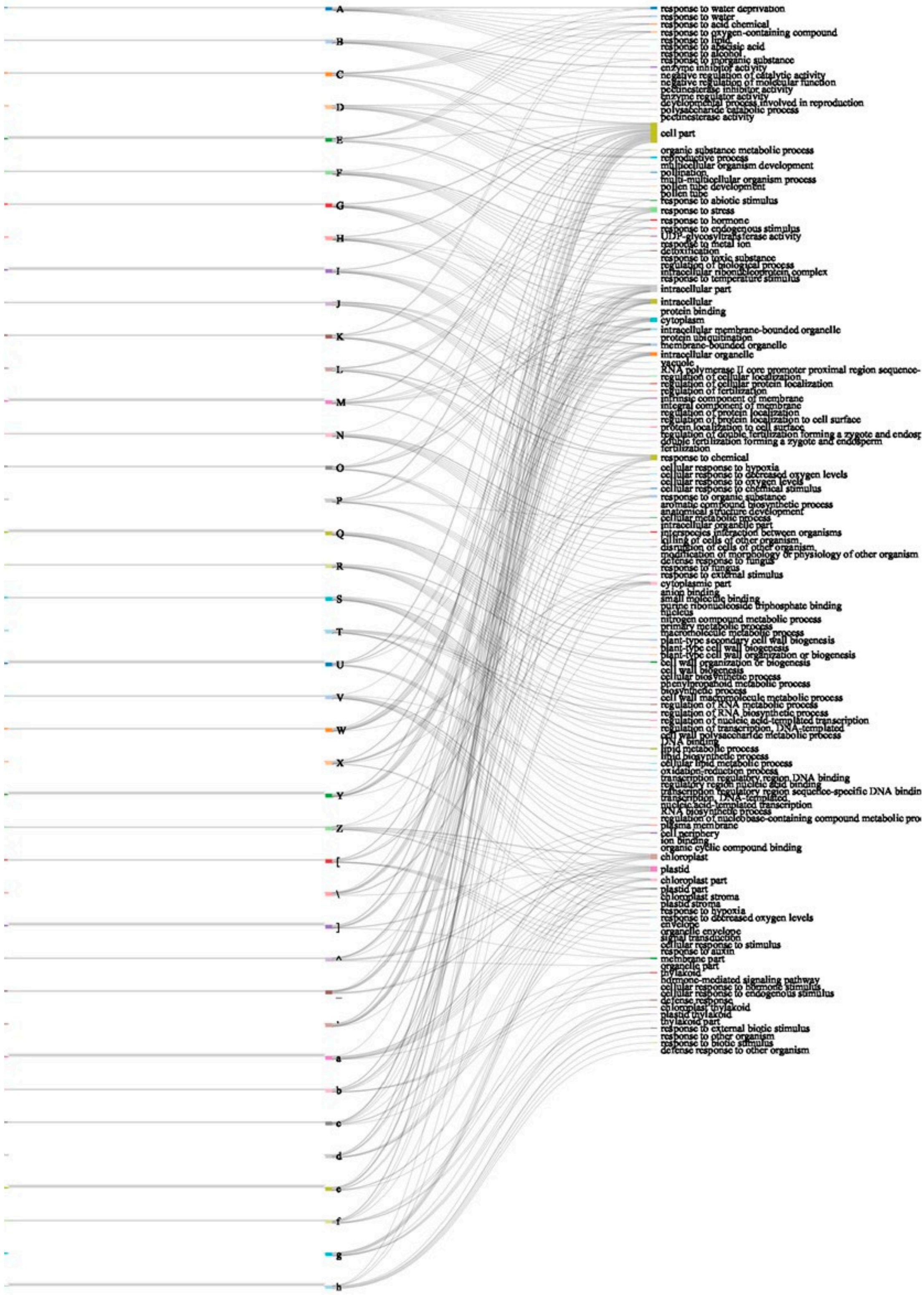

Figure S4. The iDREM analysis identified significantly enriched pathways and several TFs associated with the regulation of gene profiles in those pathways.

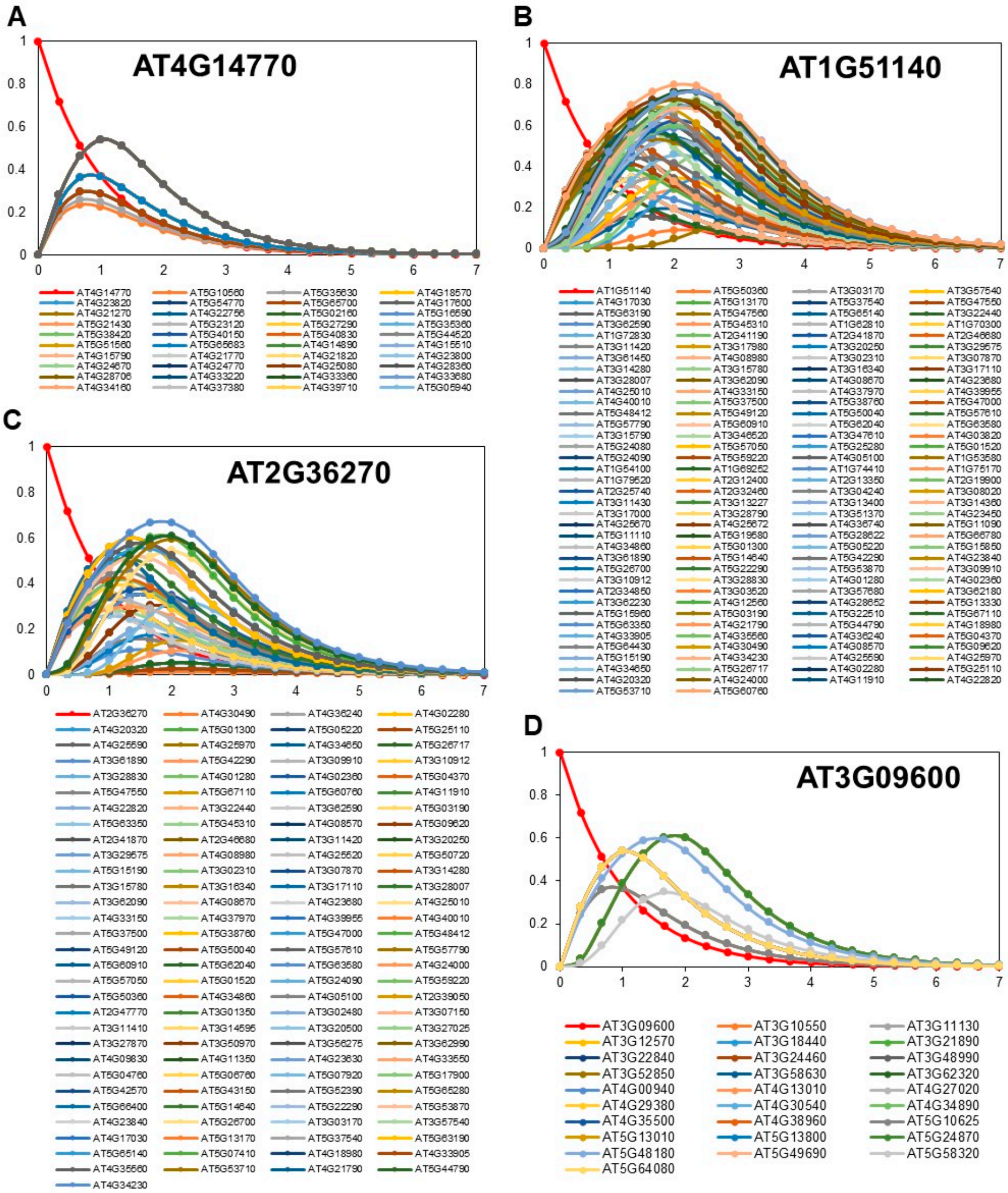

Figure S5. (A-D) The SQUAD simulation of gene activity upon the activation of TFs (AT4G14770, AT1G51140, AT2G36270, AT3G09600) in drought stress.

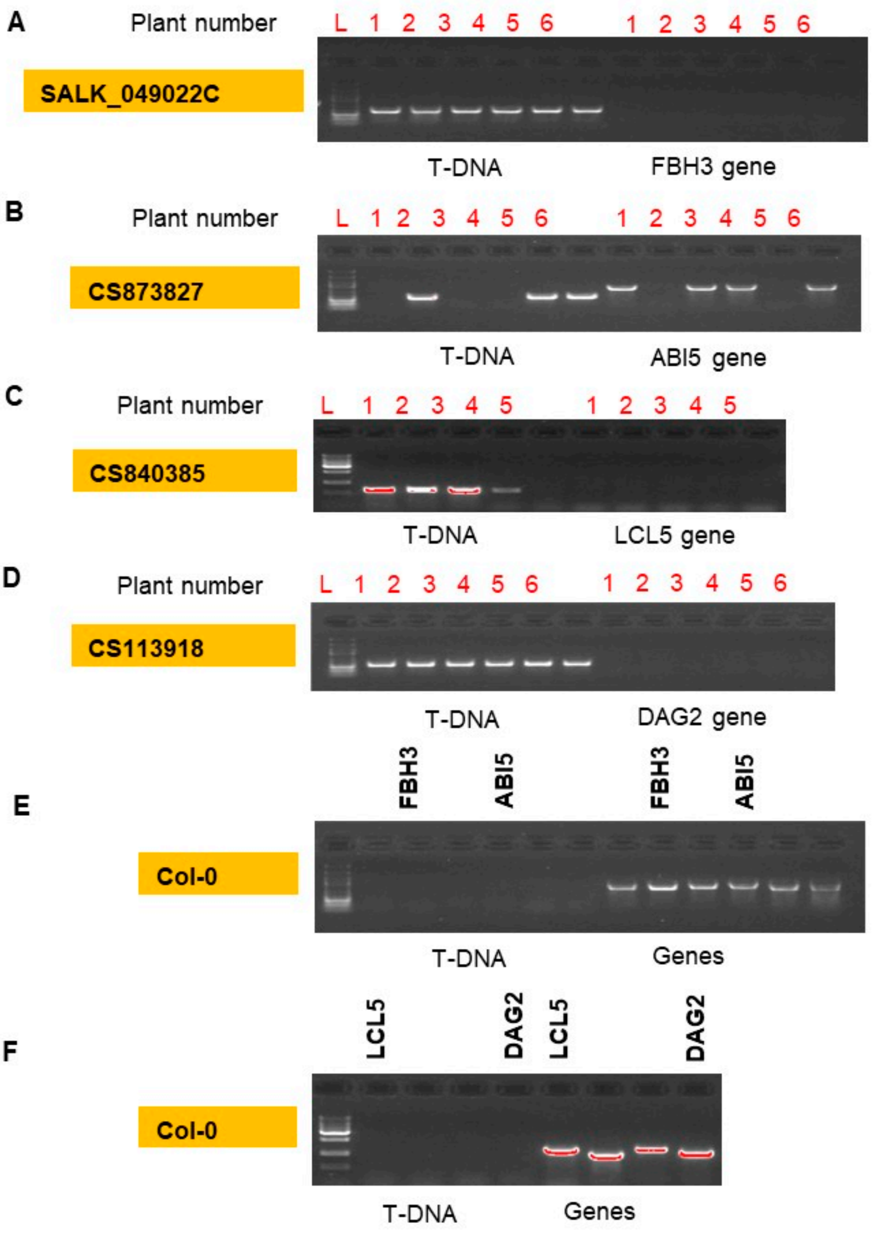

Figure S6: PCR-based characterization of homozygous mutant lines using T-DNA insert and respective gene primers for mutant A) SALK_049022C (FBH3) mutant lines B) CS873827 (ABI5) mutant lines C) CS840385 (LCL5) mutant lines D) CS113918, E) & F) Confirmation of FBH3, ABI5, LCL5 and DAG3 T-DNA insert and genes in Col-0 plants.

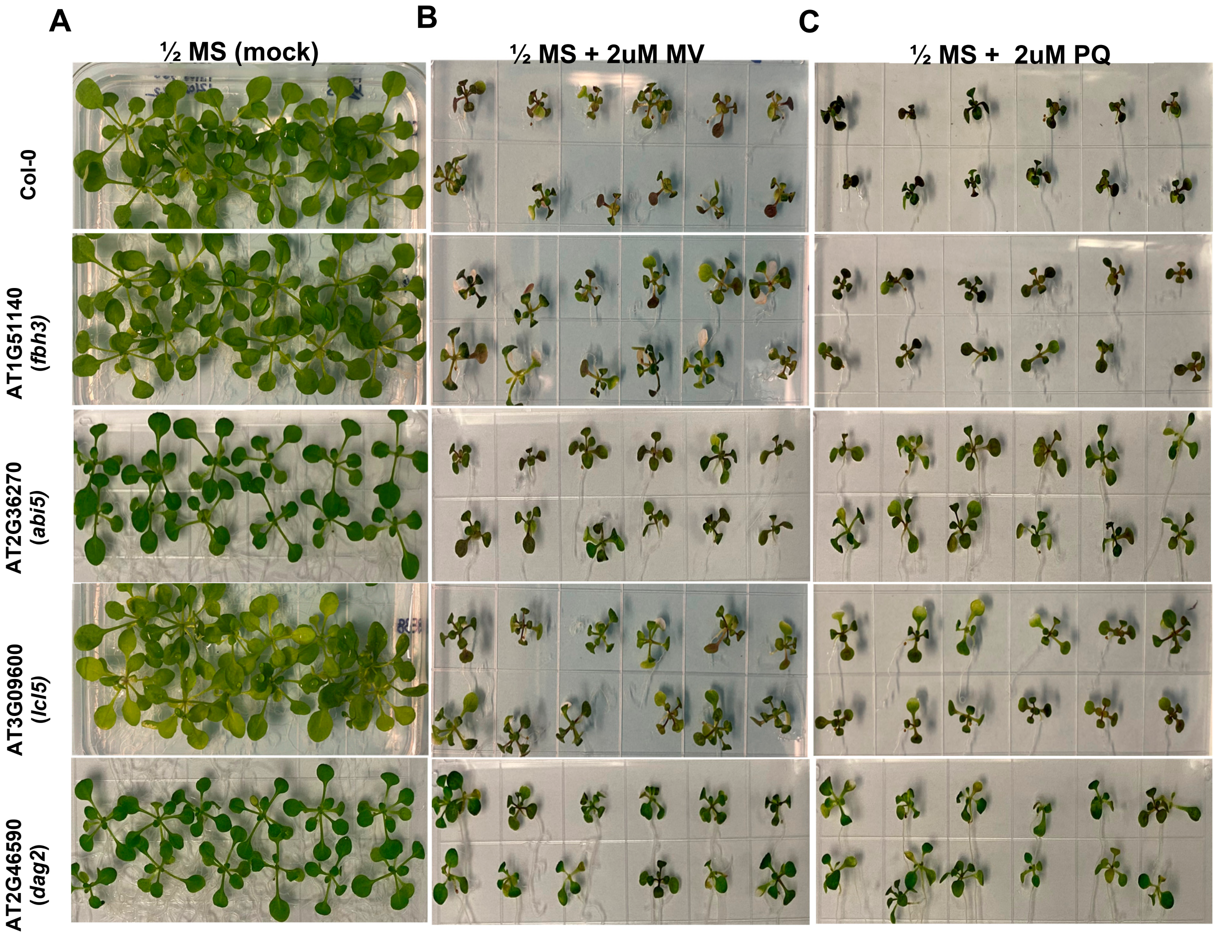

Figure S7. The visual phenotypes of with Col-0, CS840385 (LCL5), CS873827 (ABI5), and SALK_049022C (FBH3) in response to methyl viologen (MV), and paraquat (PQ)

Supplementary Tables (uploaded as separate files):

Table S 1: Gene expression, co-expression and network centrality analyses

Table S 2: iDREM and SQUAD analyses

Table S 3: Arabidopsis transcription factors and their targets included in experimental analyses; PCR primers list.
